# The Association between the Regular Use of ICT Based Mobility Services and the Bicycle Mode Choice in Tehran and Cairo

**DOI:** 10.3390/ijerph17238767

**Published:** 2020-11-25

**Authors:** Hamid Mostofi, Houshmand Masoumi, Hans-Liudger Dienel

**Affiliations:** 1 Mobility Research Cluster, Department of Work, Technology and Participation, Technische Universität Berlin, 10587 Berlin, Germany; hans-liudger.dienel@tu-berlin.de; 2 Center for Technology and Society, Technische Universität Berlin, 10623 Berlin, Germany; masoumi@ztg.tu-berlin.de; 3 Department of Transport and Supply Chain Management, College of Business and Economics, University of Johannesburg, Johannesburg 2006, South Africa

**Keywords:** ICT-based mobility services, cycling, the active mobility mode, nonmotorized mode choices, ridesourcing, ride hailing, MENA region

## Abstract

Regarding the sharp growth rate of ICT (information and communication technology)—based mobility services like ridesourcing, it is essential to investigate the impact of these new mobility services on the transport mode choices, particularly on active mobility modes like cycling. This impact is more important in the MENA context (the Middle East and North Africa), where cycling does not constitute the main mobility mode in the modal split of most MENA cities. This paper studies the relationship between the regular use of ICT-based mobility services like ridesourcing and the tendency to cycle to near destinations. This paper contains the analysis of 4431 interviews in two large cities of the MENA region (Cairo and Tehran). This research uses logistic regression to analyze and compare the odds of cycling among regular and non-regular users of ridesourcing by considering the socio-economic, land use, and perception variables. The findings indicate that the odds of cycling among the regular users of ridesourcing are 2.30 and 1.94 times greater than these odds among non-regular ridesourcing users in Tehran and Cairo, respectively. Therefore, the regular users of ridesourcing are more likely to cycle to their near destinations than non-regular ridesourcing users in these cities.

## 1. Introduction

Nonmotorized transport modes such as biking are sustainable modes in urban transportation systems, and are reliable and effective in terms of energy use and healthiness without environmental pollution [1,2,3,4,5,6,7]. In comparison to motorized modes, such as a private car, a bicycle is a cheaper door-to-door mobility mode. Moreover, compared to walking, urban biking is 3.6 times faster [8,9] and requires less energy (35% of the walking calories) for the same travel [10,11]. At this time, traffic congestion and environmental pollution are common in many cities worldwide due to high car dependency [12,13,14]. Therefore, cycling is a practical solution to reduce CO_2_ emissions and help cities’ sustainability in economic and social aspects [15,16,17]. Therefore, the international advice is to develop the bicycle’s share in the cities’ modal splits [18,19,20,21]. However, biking is not a substantial mode for daily travel purposes in some MENA cities, such as Cairo and Tehran, which is entirely different from the cycling mode share in European cities. For example, the share of biking is less than 1 percent in the modal split in Iranian cities [22]. However, cycling constitutes around 40 percent of daily trips in bicycle-friendly cities in Europe, such as Copenhagen and Amsterdam [23]. Some studies indicate different reasons for the low cycling rate in Tehran and Cairo, such as car-oriented urban forms, topographic conditions, sociocultural attitudes (women rarely use bikes), and the lack of suitable infrastructure [24,25,26,27,28]. Moreover, ICT (information and communication technology) has considerably affected the urban mobility system, as it offers real-time trip information, sourcing, and communication instruments between service providers and users. Moreover, the ICT-based mobility services have been developed very fast, such as online ridesharing and sourcing modes, in which services information technologies are the major component. The ICTs have also changed the concepts of distance, accessibility, and individual lifestyles, which consequently have a potential influence on mobility behaviors, particularly nonmotorized mode choice [29,30,31,32]. This influence is gaining more importance in the cities where the share of nonmotorized modes is low. 

This study investigates the association between the regular usage of ICT-based mobility services such as ridesourcing and the cycling mode choice in Tehran and Cairo. The primary assumption of this research is related to the principle that the frequent usage of one mobility mode affects other mode choices [33]. We conducted 4431 face-to-face interviews in Cairo and Tehran in 2017. Among ICT-based mobility services, such as online car-sharing and bike-sharing, this study focuses only on the online ridesourcing platforms because there was no considerable online bike and car-sharing in these two cities in 2017. Ridesourcing is a door-to-door mobility service in which commuters and drivers interact through ICT and GPS platforms, such as Uber and Lyft in many western countries, “Careem” in Cairo, and “Snapp” in Tehran [34]. As commuters are able to “source” a ride from a pool of drivers by ICT-based platforms, this mobility service is named ridesourcing [35]. Passengers use their smartphone apps to book, pay, and rate the quality of the services. In the MENA countries, ridesourcing has seen a sharp growth rate among other mobility modes.

Regarding the Uber report, Egypt is the biggest market of this company in the MENA region, with 157,000 drivers and 4 million users in 2017 [36]. The first ridesourcing company in Iran was Snapp, established in 2014 with a growth rate of 70% per month, with a big network of 120,000 active drivers to give services to 0.5 million users in 2016 [37,38]. These figures show the rapid growth of these new emerging travel modes in Tehran and Cairo, and indicate a potential impact on these cities’ mode choice behaviors. 

### Ridesourcing Adaptation and Biking Mode Choice 

It is necessary to study whether ICT-based mobility services support or compete with sustainable modes like cycling, in order to evaluate their role in the sustainability of urban transport systems [39,40,41]. There is a debate around ridesourcing that it encourages commuters to shift from sustainable mobility modes, like nonmotorized modes, to car travels. Regarding the findings in the global north, the adoption rate of ridesourcing is remarkably higher among young people with higher incomes and educational degrees [42,43]. Moreover, Alemi et al. (2017) mentioned a positive correlation between ridesourcing usage and the regular use of smartphones for daily activities such as shopping, entertainment, and travel [42]. Feigon and Murphy (2018) reported that ridesourcing was used for an average travel distance of between three and six kilometers in five American cities [44], indicating its possible impact on the cycling mode choice. Alemi et al. (2018) showed that due to the ridesourcing adaptation, the younger generation decreased their nonmotorized mobility choices, such as walking and biking, more so than the older people [42]. Gehrke et al. (2019) indicated the high probability of a modal shift from walking and cycling to near destinations or under poor weather conditions to ridesourcing in Boston [45]. Becker et al. (2017) mentioned that although ridesourcing can fill gaps in the public transport network, in many situations, it decreases public transport usage and nonmotorized modes, which indicates a substitution impact in favor of car dependency [46]. Circella et al. (2018) mentioned that around 40% of ridesourcing users have decreased their walking and biking, while 10% of users have increased these nonmotorized modes in California [47]. On the other hand, ridesourcing services in some countries provide more motorized mobility options for disabled people with health issues, by employing trained drivers to help passengers with walkers, wheelchairs, and other equipment [39,48]. 

## 2. Materials and Methods 

This research includes 3 main research questions: (1) Are the socio-economic variables of the respondents who use bicycles significantly different from those who do not use this mode? (2) Is there a significant association between regular ridesourcing use and the odds of cycling? (3) what are the main subjective barriers and reasons for not cycling among regular ridesourcing users? 

We conducted a large sample size of face-to-face interviews in Cairo and Tehran in different neighborhoods to answer these three questions. Regarding the literature review about the urban forms in Cairo and Tehran, the compactness, population density, urban forms, and road network forms in these two cities have correlations with the periods of urban construction and development [49]. The newly developed neighborhoods are centerless and have less population density and compactness than older neighborhoods in Tehran and Cairo. As such, we chose six neighborhoods in each city in three different urban forms (two neighborhoods in each urban form) to gather adequate samples. These three different urban forms are traditional parts (historical), transitional parts (in between), and newly developed parts. The traditional (historical) neighborhoods in both cities are discernible center with high density and compact urban forms. Transitional neighborhoods were constructed from 1930 till 1980 with lower density and compactness than the old parts. The new parts, which were developed after 1980, are centerless neighborhoods and are located in the peripheral parts of Tehran and Cairo. The interviewers conducted face-to-face interviews with the residents of these neighborhoods who were selected randomly in 2017. The total observations are 4431 interviews for these two cities, including 2369 interviews in Tehran and 2062 interviews in Cairo.

Many studies reported that the cycling mode choice has an association with age [49,50,51], socio-economic parameters, road network and land use parameters [52,53], the safety of cycling paths with physical separation from the motorized network [54], efficient facilities [55,56], and the gently graded topography of cities [57]. Therefore, we designed three main sections in the questionnaire, which are (1) socio-economic variables, (2) mobility behavior variables, and (3) the land use parameters of the neighborhood. The socio-economic factors included gender, age, monthly household expenditure and income, car ownership, and having a driving license. The variable of car ownership is a binary variable, which indicates whether the household has at least one car or not. The variable of possession of a driving license is also a binary variable, indicating whether the respondent has a driving license or not. The economic factors of income and monthly expenses were assessed in Egyptian pound (the Egyptian currency) and Iranian rial (Iranian currency). 

### 2.1. The Mobility Behavior Variables

The mobility behavior section includes questions in two main subsections, which are the main modes for common daily travel purposes and the tendency towards cycling to near destinations. The regular trip purposes include study or work trips and non-work/study trips within and out of the respondents’ neighborhoods. We defined a binary variable, “ridesourcing use”, to study the impact of ridesourcing adaptation as the main mode on the tendency to cycle. This variable categorizes the interviewees into 2 groups: (1) regular users; (2) non-regular users. The regular users of ridesourcing are defined as the commuters who frequently use ridesourcing as the major mobility mode for a minimum of one of their daily trip purposes. The non-regular users are the commuters who do not use and adopt ridesourcing as their major mobility mode for their everyday trip purposes. Therefore, “ridesourcing use” is a dichotomized variable that is collectively exhaustive and mutually exclusive. Therefore, this variable classified all observations of the Tehran and Cairo samples into 2 classes, and these two classes do not have the same observation. 

In the second subsection, the respondents answered whether they cycle to a near destination inside their neighborhood. This question aims to capture the tendency of respondents to cycle inside their neighborhoods to a destination that is near based on their subjective perception of distance. Therefore, in this question, we did not mention the distance in meters or kilometers. We mentioned the term of “near destination” to understand whether the respondent tends to cycle even to a destination that he/she perceives as a near destination. The dichotomized variable is defined as “bicycle use for a near destination,” coded 1 and 0 for the yes and no responses, respectively. Additionally, the respondents were asked about their main reason for not cycling to near destinations. The respondents could only choose one major reason for the multiple-choice question. These options were designed in the present form to collect subjective reasons for not cycling. This question asks for the interviewees’ perceptions about cycling to a near destination. We designed the answers to this multiple-choice question following the review of research and reports about cycling barriers in the MENA cities [58,59,60,61,62,63]. The options include four factors, which are (1) cultural and social problems, (2) lack of biking facilities, (3) disabled/too old, (4) takes too much time/it is slow. 

### 2.2. The Land Use Parameters of the Neighborhood

Regarding the role of the land use and road network factors in the tendency to cycle, two parameters were measured, indicating the neighborhoods’ connectivity. These parameters are “link–node ratio (%)” and “intersection density”. Link–node ratio (%) is the number of links (street segments) divided by nodes (street intersections) within the 600 m catchment of every interviewee’s house. A bigger link–node ratio indicates the greater connectivity of the road network in the interviewee’s neighborhood. This parameter illustrates how many possible routes there are in the neighborhood per each node for cycling. However, the link–node ratio is unrelated to the size of the intersections or blocks [64]. Intersection density (nodes/ha is the sum of intersections per unit area in a 600 m catchment area of the interviewee’s house) is related to the size of blocks in a neighborhood [65]. The bigger intersection density suggests more connectivity in a neighborhood because of the smaller blocks and the shorter cycling distances inside this neighborhood. The full details of the neighborhood characteristics in this survey were published [66].

### 2.3. Analysis Methods

#### 2.3.1. Comparison of the Demographic Variables 

We compared the socio-economic parameters of two groups of respondents who use bicycles and who do not use this mode for a near destination in the samples of Cairo and Tehran. First, the Kolmogorov–Smirnov test was applied to check whether the distribution of the continuous socio-economic variables is normal. The result indicates a *p*-value less than 0.001 for the variables of age, monthly household income, and monthly household living costs, indicating their distributions are not normal. Therefore, we applied nonparametric tests, such as the Mann–Whitney U test and the median test, to evaluate whether the differences in the distribution and median of the mentioned continuous variables are significant across the binary variable (bicycle use) at the confidence level of 95%. The H_0_ of the Mann–Whitney U test indicates that the distribution of each socio-economic variable is the same between two values of the binary variable “bike use”. The H_0_ of the median test assumes that the continuous variable has a similar median between two groups of respondents who ride a bicycle and do not ride it for a near destination. Regarding gender and household car ownership variables, the hypotheses are defined for each variable about the significant correlation between bicycle use and gender variables. We applied the Chi-square test to test this hypothesis at a confidence level of 95%. 

#### 2.3.2. Association between Frequent Ridesourcing Use and Odds of Cycling 

We used binary logit models (logistic regression) to compare the probability of cycling between regular and non-regular users of ridesourcing in each sample of these MENA cities at significance levels of 0.05. The odds of bicycle use for a near destination are the probability of cycling over the probability of not cycling, which are response variables in the logit models. The transformation from probability to odds is monotonous, indicating the odds increase (decrease) as the probability increases (decrease). The binary logit model is structured by the equations below, where P is the probability of cycling to a near destination, 1 − *P* is the probability of not cycling, *β*_0_ is the constant, and *β_i_* is the coefficient related to each explanatory variable.

(1) $$ln\left(\frac{P}{1\;-\;P}\right)=\beta_{0}+{\;\beta }_{1}x_{1}+{\;\beta }_{2}x_{2}+{\;\beta }_{3}x_{3}+\;\ldots \;+\beta_{n}$$

(2) $$P=\;\frac{e^{(\beta_{0}+{\;\beta }_{1}x_{1}+\;\ldots \;+\beta_{n}x_{n})\;}}{1+{\;e}^{(\beta_{0}+{\;\beta }_{1}x_{1}+\;\ldots \;+\beta_{n}x_{n})\;}}$$

The logistic model reveals the effects of the independent variables on the odds of bike use (probability of bike use/probability of no bike use). The exponentiated coefficient changes the cycling odds to a unit increase in the independent variable by holding other regressors constant. Suppose the odds ratio of an independent variable is greater than 1. In that case, it is suggested that by keeping other regressors constant, the odds of bike use increase (decrease) by increasing (decreasing) this independent variable. If the odds ratio is 1 for an estimator, it suggests that this estimator’s change does not change the cycling odds. When the odds ratio of one estimator is less than 1, it indicates a decrease in biking odds caused by an increase in this estimator if other independent variables are constant. Some transport studies estimate the odds ratio to show the impacts of the explanatory variables on the odds of mode choices. However, the estimation of average marginal effects is an intuitive technique and a useful way to directly explain the probability changes and interpret the results of the estimations more understandably. The logistic regression is nonlinear; therefore, the effect of one unit change in an explanatory variable is averaged over all observations to estimate the average marginal effects. The average marginal effects of an independent variable are the average change in probability of the dependent variable when the given independent variable changes by one unit and the other is constant. We used the add-on package “margins” in R to calculate the average marginal effects. Therefore, in addition to the odds ratio, we also report the average marginal effects of each explanatory variable.

We checked the risk of multicollinearity among the independent variables of one pair or more of explanatory variables being highly correlated together and causing unreliable estimations. The regressors were selected to avoid high multicollinearity among variables and to control confounding effects. Therefore, the regressors (independent variables) in the logistic regression are regular ridesourcing, household car ownership, gender, age, monthly household income, intersection density, and link–node ratio. Checking the correlation matrix might be useful in order to detect multicollinearity, but it is not enough. The sufficient diagnostics are performed by linear regression between the variables to check the variance inflation factor (VIF), tolerance, and the condition index. If the correlation coefficient among two independent variables is greater than 0.90, it indicates a high multicollinearity risk in the logit model [67,68]. We checked the correlation matrix, and we did not find a correlation greater than 0.8 for both samples of Cairo and Tehran. Furthermore, we performed the linear regression among the different combinations of explanatory variables and then checked the VIF, tolerance, and condition index. The VIF for an estimator in the regression model is the ratio of the variance of the overall model to the variance of a model that includes only the given estimator. Hair et al. (2010) indicated that if the VIF is greater than 5, there is a concern for multicollinearity among independent variables [68]. As we checked the VIFs for different combinations of the estimators, the VIFs of all estimators in the combination of regular ridesourcing, household car ownership, gender, age, income, intersection density and link–node ratio are less than 4.0 in both the Cairo and Tehran models. Then, we checked the tolerances of the mentioned variables, which is the amount of variability in one estimator that is not explained by the other estimators. Tolerance values less than 0.2 indicate the risk of multicollinearity among estimators. Having checked the tolerance values for the mentioned independent variables, all of them were bigger than 0.3, indicating the low risk of multicollinearity. Moreover, we checked the condition indices. Condition indices above 15 indicate a risk of multicollinearity. The estimated condition indices were below 15 in the linear regressions among the aforementioned estimators. Moreover, we checked the interactions between the variables, such as monthly household income–age, monthly household income–gender and gender–age, in the two logit models of Cairo and Teheran, and estimated their coefficients. The coefficients of the interaction terms between these variables were not significant at the 95% confidence level in the models of both cities. For example, the coefficient of the interaction age–monthly household income has a Wald chi-square = 2.610, and a *p*-value = 0.106 in the Tehran model, and a Wald chi-square = 0.364 and *p*-value = 0.547 in the Cairo model. Therefore, we did not consider the interaction terms between the variables in either model. We applied the Omnibus test to check if the regression model with estimators is an improvement of the baseline model without estimators. Moreover, the Hosmer and Lemeshow test was applied to check if the regression result was correctly specified, and the results were appropriately fitted to the observed data. If the *p*-value of the Hosmer and Lemeshow test is less than 0.05, it suggests a significant difference between the observed values and the model’s estimated values [69]. 

## 3. Results

### 3.1. Demographic Profile 

Among the 2062 interviews in Cairo, 224 respondents mentioned that they use a bicycle for a near destination, and this result for Tehran is 254 out of 2369 interviews. Table 1 shows the socio-economic parameters, including age, gender, household income, household living cost and household car ownership, for the two groups of respondents who use and do not use a bike for a near destination in Tehran and Cairo. 

The Chi-square test suggests significant associations (*p* < 0.001) between the variables of “bicycle use for a near destination” and household car ownership and gender in the Cairo sample. This means that men are substantially more likely to cycle to a near destination than women in Cairo. Moreover, 58.5% of bike users and 70.5% of non-bike users have at least one household car, which indicates a different rate in car ownership among users and non-users of bikes in Cairo. Furthermore, the Mann–Whitney U test indicates a significant association between bike use and age at the 99% confidence level. Furthermore, the median test suggests that the medians of age are different across bike users and non-users for a near destination at the 0.05 level (*p* < 0.05). The mean and median of bike riders’ age are 25.6 and 24.0, respectively, which indicates they are significantly younger than non-bike riders in the Cairo sample. 

The medians and the Mann–Whitney U tests do not reject the H_0_ (null hypotheses) for the variables of monthly household income and living costs across variables of bike use in the Cairo sample. This means that there are no significantly different distributions and medians in monthly household living expenses and income between bike users and non-users.

In the Tehran sample, the Chi-square test indicates a significant association at the 0.001 level among the variables of gender and bike use. This suggests that men cycle considerably more than women in Tehran. The Mann–Whitney U test rejects the null hypothesis for age at the 99% confidence level, which is similar to the result of this test in the Cairo sample. Furthermore, the median test reveals that the median age is significantly different across users and non-users of bikes. Therefore, the respondents who tend to use bikes to travel to a near destination are significantly younger than those who do not have this tendency. Moreover, these tests do not reject the H_0_ for the variables of monthly household income and living expenses across users and non-users of bikes at the 0.05 level in the Tehran sample. Therefore, like the Cairo sample, these two tests indicate that the monthly household living costs and income are not significantly different between the users and non-users of bikes. 

### 3.2. The Logit Models for Cycling to a Near Destination 

The logistic regression model is used to compare the odds of bike use between regular ridesourcing users and the non-regular users for each sample of Cairo and Tehran. For the categorical variable of ridesourcing use, we defined the non-regular ridesourcing user as the reference mode in the logistic regression. The Omnibus test shows a significant difference at the 0.001 level between the log-likelihoods of the model with estimators and the baseline model (without estimators), with Chi-square values of 233.955 and 274.906 for the samples of Cairo and Tehran, respectively. 

The Hosmer and Lemeshow test indicates that the goodness of fit for both the Cairo and Tehran models is appropriate. The *p*-value of this test is 0.773 for the Cairo sample and 0.092 for the Tehran sample. Table 2 shows the Omnibus test results, the Hosmer and Lemeshow test, and the Nagelkerke R squared for the logistic regressions. The coefficients of estimators are shown in Table 3 and Table 4 for Tehran and Cairo samples, respectively.

Tehran’s model suggests three significant variables with a *p*-value < 0.001 level—gender, age, and link–node ratio. The model also indicates that the ridesourcing use and intersection density variables are significant at levels 0.05 and 0.01, respectively. As “ridesourcing use” is a binary variable, its exponentiated coefficient shows the odds ratio of cycling for regular users relative to the non-regular ridesourcing users. The odds of cycling for the regular ridesourcing users are 2.30 times more than these odds for non-regular users, when fixing all other regressors as constant. The average marginal effects of ridesourcing use indicate that being regular users of ridesourcing services increases the probability of cycling to a near destination by 6.0%. Therefore, the results reveal that regular ridesourcing users are more likely to cycle to a near destination than non-regular users. The marginal effects of gender indicate that the cycling probability of women is 14.9% less than men. Moreover, the model suggests that the odds of biking for women are 87% less than for men with the same other independent variables. Each unit increase of link–node ratio (percent) raises the odds of cycling by 2.5% and the cycling probability by 0.2%. Each unit increase in intersection density (node/hectare) increases biking odds by 13.8% and biking probability by around 1%. Therefore, Tehranians living in a neighborhood with better intersection density and node–link ratio are more likely to cycle to a near destination. The model of Tehran indicates that the odds ratio and average marginal effects of age are 0.94 and −0.004, respectively. Therefore, a one-year increase in age decreases the biking odds by 6% and the biking probability by 0.4%. This means younger citizens are more likely to bike than older citizens in Tehran.

Table 4 illustrates the binary logit regression for Cairo with three statistically significant variables at the significant level of 0.001: household car ownership, gender, and age. Furthermore, the variable ridesourcing is significant at the 0.05 level. The model shows that the cycling odds for the regular ridesourcing users are 1.94 times greater than non-regular users by controlling for other independent variables. Moreover, the average marginal effects of regular ridesourcing are 5.3%. This result suggests that regular users are more likely to cycle to a near destination than non-regular users of ridesourcing. This result is similar to the Tehran sample, whereby regular users of ridesourcing are also more likely to use bikes than non-regular users. Like the Tehran model, the average marginal effects of gender are −15.4%, and the odds of biking for women are 86% less than these odds for men, with the same values for the other regressors. Moreover, the people who do not have a household car have biking odds 2.44 times and a biking probability 7.0% greater than those who have at least one household car. The model indicates that the odds ratio of biking and the average marginal effects of age are 0.92 and −0.006, respectively. The increase of one year in age decreases the cycling odds by 8%, and its probability by 0.6%. Therefore, older citizens are less likely to cycle than younger ones. Moreover, the model suggests that the coefficients of the variables income, intersection density, and link–node ratio are not significant at the 95% confidence level. Therefore, the binary logistic regression results reveal that these variables do not contribute to the estimation of the odds and probability of cycling to a near destination, and the other variables have a more significant influence on the tendency to cycle in the Cairo sample.

### 3.3. Reasons for Not Cycling

**Figure 1:**
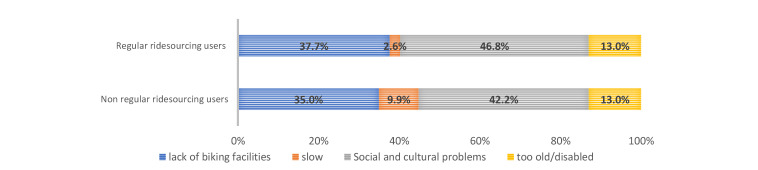
Reasons for not cycling among regular and non-regular ridesourcing users in the Tehran sample.

**Figure 2:**
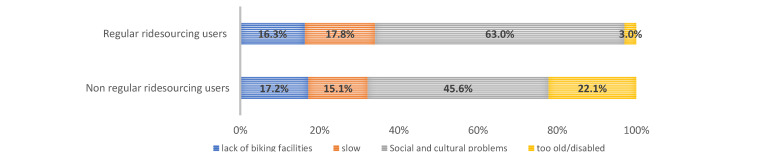
Reasons for not cycling among regular and non-regular ridesourcing users in the Cairo sample.

**Figure 3:**
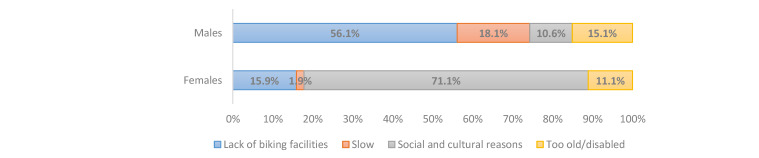
Reasons for not cycling among female and male respondents in the Tehran sample.

**Figure 4:**
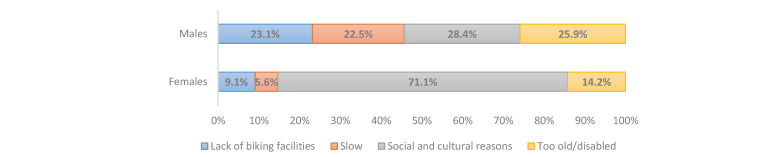
Reasons for not cycling among female and male respondents in the Cairo sample.

We asked about subjective barriers to and reasons for not cycling to a near destination in the interviews in Cario and Tehran. The form of this question is multiple-choice, but the respondents should choose only one reason out of the four reasons, which are (1) cultural and social problems, (2) lack of biking facilities, (3) being disabled or too old, and (4) it is slow or takes too much time. We asked this question in the present form to collect subjective reasons correlated with the way citizens decide about not cycling to near destinations in their neighborhoods. The subjective reasons are usually highly correlated together and constitute a package of factors for mode choice. However, we asked respondents to choose one reason in order to find the dominant and major subjective obstacle to cycling. This question type might be useful for urban planners to understand which problems are more influential on the tendency of citizens toward cycling. For example, suppose the cycling facilities in a neighborhood are improved. In that case, the person who mentioned “lack of biking facilities” as the main reason for not cycling is more likely to cycle than the person who replied “being disable or too old” as the main reason for not biking. Social and cultural problems address social barriers to cycling for women in public spaces, inconvenience during cycling in public areas, and fear of harassment. The answer “lack of biking facilities” indicates the lack of bicycle infrastructures like bike lanes, excluding motorized modes in the road network and public shared bikes. The findings are presented in Figure 1 and Figure 2 for regular and non-regular users of ridesourcing in both cities. Figure 3 and Figure 4 illustrate the reasons for not cycling among female and male respondents in Tehran and Cairo.

The results of this question in the Tehran sample indicate that the major reasons for regular ridesourcing users not cycling are “social and cultural problems” and “lack of cycling facilities”, which 46.8% and 37.7% of regular users mentioned, respectively. These two reasons were also selected more than the other reasons by the non-regular users in this sample. This finding indicates that the perceptions of social and cultural problems and the lack of cycling facilities are the main reasons for regular and non-regular ridesourcing users not cycling to a nearby destination in Tehran. Moreover, Figure 3 shows that 71.1% of female respondents who do not cycle indicate the social and cultural problem as their main reason for not cycling in Tehran. However, 10.6% of male respondents mentioned the social and cultural problems as their major reason for not cycling. Therefore, Figure 3 shows that the social and cultural problems are substantial barriers for women wishing to cycle in Tehran.

In Cairo, the major reasons of regular ridesourcing users for not cycling are “social and cultural problems” (63%), “It is slow/takes too much time” (17.8%), and “the lack of biking facilities” (16.3%). The non-regular users reasoned “social and cultural problems”, “too old or disabled”, and “the lack of biking facilities”, with 45.6%, 22.1%, and 17.2%, respectively.

Figure 4 indicates that 71% of female respondents in the Cairo sample mentioned the social and cultural problems as their main reason for not cycling, while 28.4% of males cited this option as their major reason. Therefore, as in the Tehran sample, social and cultural problems are significant barriers against women cycling. Moreover, the reason “the lack of biking facilities” was mentioned by the regular users in Tehran almost two times more than these users in Cairo. Furthermore, 13% of regular users in Tehran stated the reason “too old/disabled,” which is noticeably more than Cairene regular users (3%), and is the lowest observed reason in the Cairo sample.

## 4. Discussion

We used large samples in Cairo (2062 interviews) and Tehran (2369 interviews) to study the effect of the frequent use of ICT-based mobility services on the tendency toward bike use. The findings show that a low percentage of the respondents use bikes for a near destination, which is 10.9% in Tehran and 10.7% in Cairo. This result confirms the other research’s conclusion that bicycles are not a substantial mobility mode in Tehran and Cairo [22,25,26,28,70,71]. Therefore, our findings show that bicycles’ share in the modal split of these cities is lower than in most western countries, such as in Europe [23,24,72]. As such, our findings emphasize the necessity of raising public awareness about the social and health benefits of cycling, as well as the improvement of the bike facilities to encourage citizens to use this mobility mode more.

### 4.1. The Relationship between the Tendency toward Biking and Frequent Ridesourcing Use

Regarding the growing share of ICT-based mobility modes, such as ridesourcing, it is necessary to investigate the impact of these new emerging transport services on mobility behaviors. This effect on the bike mode choice is particularly important in the MENA region, which has a low share in the cities’ modal mobility split. However, the studies in this field often come from western countries, while there is little research in developing countries like the MENA region. Moreover, with the emergence and rapid rise of ridesourcing in the modal split of MENA cities, it is essential to analyze the impacts of this new mobility mode on people’s mode choice behaviors in this region. Cairo and Tehran are the two largest cities in this region, with similarities in urban development in recent years. The development of urban forms in both cities is more car-oriented rather than sustainable mobility modes-oriented. Moreover, transportation network companies launched their services in a similar period with the same growth rate. Therefore, we studied the impacts of ridesourcing on citizens’ bike mode choices in these two cities as a case study in the MENA region. Among ICT-based mobility services, such as ridesourcing, online car and bike sharing, we focused on ridesourcing to study the correlation between the regular use of this mobility mode and bike use for near destinations in Tehran and Cairo. Other ICT-based mobility modes have not been developed enough to be considered in these two cities’ modal splits. Some western studies investigated the modal shift from active mobility modes like cycling to ridesourcing by counterfactual questions in their interviews and questionnaires [35,42,43,44,73,74]. These counterfactual questions ask, “what would you have done if ridesourcing services had not been available?”. The interviewees should think about a manipulated memory to answer this kind of question. However, this study categorized the respondents into the two groups of regular ridesourcing users who adopt ridesourcing for their everyday trip purposes and non-regular ridesourcing users. Then, we studied their current tendency toward cycling to a near destination via the question framed in the present tense, intended as the think-forward question, “Do you use a bike for a near destination?”. Thus, we analyzed the association between the frequent use of ridesourcing and present tendency towards cycling, instead of the past modal shift among these mobility modes.

We applied binary logistic regressions to analyze the association between frequent ridesourcing use and the odds of biking to near destinations. The results revealed that the odds of cycling for the frequent ridersourcing users are 2.30 (in Tehran) and 1.94 times (in Cairo) more than these odds for non-regular users. Furthermore, the average marginal effects of being a regular ridesourcing user are 6.0 and 5.3% in the models of Tehran and Cairo, respectively. This means that in these cities, people who have adopted ICT mobility modes, such as ridesourcing, as their major mobility mode are more likely to cycle to a near destination than the non-regular users of ridesourcing. This result is in contrast to some western studies that suggest frequent ridesourcing use has a negative association with biking. For example, 40% of regular ridesourcing users in Californian reported that they decreased cycling [47].

Moreover, the studies in American cities reported a substitution of active modes, such as biking, in the range of 9% and 13% of total ridesourcing trips in San Francisco, Boston, and Denver [35,45,73]. Moreover, a similar study in Chinese cities reported that 6.5% of ridesourcing users reduced walking and biking in the Chinese cities [75]. The finding of this paper, that in Tehran and Cairo, where cycling does not constitute a considerable travel mode, the adaptation of ridesourcing has a positive association with the likelihood of cycling, is important. It shows that the regular users of ridesourcing who adopt information technologies in their mobility modes tend toward cycling more than non-regular ridesourcing. Therefore, this finding indicates the potential for online-based bike-sharing services in Tehran and Cairo because the people who regularly use online-based ridesourcing also might use more online bike-sharing for their near destinations in these two cities.

### 4.2. Socio-Economic Variables

The associations between the socio-economic parameters and bike use for a near destination were studied in Tehran and Cairo. The findings indicate that women are significantly less likely to cycle to a near destination than men in both cities. Other transport studies also confirm this result in the MENA region, finding that women do not use bicycles because of cultural and social problems [71,76,77,78]. However, in the high cycling countries, such as Belgium and the Netherlands, women within the 20–65 age group use bicycles more than men [79,80]. Moreover, the findings indicate that younger people tend to use bikes more than older citizens in both cities, which is similar to the findings of studies in western countries such as the UK [81,82] and the MENA region [78]. Although many studies indicate the important role of cycling and its health benefits for the older ages [65], our findings showed that older people have a significantly lower tendency to cycle. Although the association between biking and aging might be negative in higher cycling countries, the share of cycle traveling in the older age groups is high. For example, cycling decreases with aging in Denmark, but still, among people who are 70–74 years old, cycling constitutes 12% of all their travels, which is similar among Germans who are 65 and older. Moreover, Dutch people over 65 years old make 24% of all their trips by bike [65].

Additionally, the logistic regression results indicate that Cairenes who have at least one household car are significantly less likely to use a bike for a near destination than those who do not have household cars. This finding is similar to studies in some American cities [83]. However, the Tehran model does not suggest a significant association between the probability of biking and car ownership, which is similar to the findings of Fishman et al. (2013) and Carse et al. (2013) [84,85]. In addition, the logit models of the Tehran and Cairo samples do not indicate a significant coefficient for the variable of income at the 95% confidence level. Moreover, the Mann–Whitney U and median test results do not show significant differences in the distributions and medians of monthly household incomes and living expenses among users and non-users of bikes. Therefore, these findings indicate that a tendency towards cycling does not have a significant association with the incomes of citizens in Cairo and Tehran.

### 4.3. Impact of Land Use Parameters

We used two land use parameters to analyze the correlation between the connectivity of neighborhoods and the tendency towards cycling among regular and non-regular users of ridesourcing. Tehran’s binary logit model reveals that the connectivity variables have significant odds ratios bigger than 1, which are 1.138 and 1.025 for intersection density and link–node ratio, respectively. In other words, by improving the connectivity of the neighborhoods, Tehranians are more likely to cycle to close destinations inside their neighborhoods. This result is the same in the related studies in both the western context [86,87,88,89] and the MENA context [56,90], which indicated that increasing the connectivity of neighborhoods increases the nonmotorized mode choices, such as bicycles, which promote physical activity. However, these land use variables do not have significant odds ratios in the Cairo logit regression. As such, the Cairo model shows that the probability of biking to a near destination is more associated with the other parameters, such as gender, age, and household car ownership. In other words, because of the impacts of the other factors, the probability of cycling in neighborhoods with good connectivity is not significantly different from the neighborhoods with low link–node ratio and intersection density. This finding is similar to some studies in the western context [60] and the MENA cities [91,92], indicating that other parameters such as social and cultural factors have more substantial effects than land use factors (i.e., connectivity) on nonmotorized mode choices such as cycling.

Moreover, some studies indicate the important role of terrain slope and the elevation profile of cities in their citizens’ cycling behaviors and mode choices [93,94,95]. The average terrain slope, which is calculated in percentage, has a negative correlation with the tendency to cycle [96]. If the slope of a road is more 3%, it is not convenient to cycle and control bikes, and consequently, the citizens are less likely to cycle in this condition. [97]. In Tehran, the slope direction decreases from the north to the south, which means the ground height reduces gradually from the north to the south of Tehran. Regarding the topographic studies in Tehran, most areas have a slope between 0 and 3%, and some small residential districts in the north of this city have a slope of more than 3% [97]. Therefore, for most parts of Tehran (except some districts in the north part), the steep slopes of roads are not major barriers for cycling. Two high plateaus surround Cairo in the east and the west. According to the topographic studies in Egypt, Cairo’s average terrain slope is below that of the most bicycle-friendly cities in the world [98]. Therefore, the average terrain slope of the neighborhoods does not make a problem for cycling in Cairo.

### 4.4. Reasons for Not Cycling

The most observed reasons for Tehranian regular ridesourcing users not cycling are “social and cultural problems” and “lack of biking facilities”. In the Cairo sample, the main excuses of regular ridesourcing users for not cycling include “social and cultural problems”, “It is slow/takes too much time”, and “the lack of biking facilities”. The social problems are the most observed reason in both cities, which refer to social barriers to women cycling in public spaces in the MENA countries, inconvenience in public areas, and fear of harassment. Davies et al. (2001) also reported social and cultural problems as the main barrier against cycling in some British cities in 2001 [99]. However, the problems women face when cycling are the dominant social barrier in Tehran and Cairo. Regarding this barrier in the MENA region, it is necessary to raise public awareness about the health benefits and positive environmental impacts of cycling, and encourage women to use bikes through cultural programs.

### 4.5. The Necessity for Improvement in Biking Infrastructure

The lack of cycling infrastructures is the second and third most observed reason for not cycling among regular ridesourcing users in Tehran and Cairo, respectively. Some studies indicate the important role of appropriate biking infrastructure in raising the tendency toward biking. Dill and Carr (2003) indicated that improving biking infrastructure is associated with increasing bike use [100]. Moreover, developing special bicycle facilities, such as bike lanes and adding traffic lights for cycling, decreases the risk of crashes and injuries and significantly improves cycling safety in cities, which consequently encourages citizens to bicycle to their destinations more often. Therefore, these cities need to improve the cycling infrastructure by increasing the special bike lanes, city bikes, and bike-sharing services. The findings of this section confirm the suggestion of other similar studies, that increasing cycling among citizens needs to be addressed by infrastructural improvements and changes in social and individual perspectives on cycling [101,102,103,104].

### 4.6. Limitation and Further Research

In this paper, we studied the subjective reasons for not cycling in Tehran and Cairo. The findings indicate the social and cultural problems as the major barriers to cycling in both cities. Therefore, we suggest further qualitative and quantitative research into the impacts of social and cultural problems on cycling in more detail, in order to distinguish the different social aspects in this field. Moreover, in this study, we did not access the precise data of slope roads around the houses of each of the respondents. Therefore, further research is proposed to study the impact of different road slopes among the neighborhoods on citizens’ tendencies to cycle in Tehran and Cairo. Moreover, we propose further research to gather more detailed data of the physiological conditions related to respondents’ age, so as to develop the models of bicycle mode choice in these two cities, with more explanatory variables and their interactions.

## 5. Conclusions

This research studied the relationship between the regular use of ICT-based mobility services such as ridesourcing and the biking mode choice in the MENA region (Tehran and Cairo). Its findings suggest that although cycling does not have a considerable share in Tehran and Cairo’s modal split, the frequent ridesourcing users are more likely to use bicycles than non-frequent users. This finding shows the potential for online bike-sharing in both cities where this online mobility mode has not been developed considerably in recent years. Following this, the people who regularly use online-based ridesourcing also might use online bike-sharing more often for their near destinations in these two cities. Moreover, the findings indicate that women and elderly citizens are significantly less likely to cycle to a near destination than men in both cities.

Furthermore, the built environment parameters of the neighborhood, such as intersection density and link–node ratio, have a significant positive correlation with the probability of biking in Tehran. The reasons for not cycling are studied among regular ridesourcing users, and the results show that social and cultural problems are the main barriers to the use of bicycles in both cities. Therefore, our findings suggest developing a new shape of online bike-sharing, raising awareness of the health and environmental benefits of cycling, particularly among women and older people, as well as improving bike facilities to promote the share of cycling in Tehran and Cairo.

## Figures and Tables

**Table 1:** The socio-economic parameters in the Tehran and Cairo samples.

Do You Use Bicycle for a Near Destination?		Tehran				Cairo			
		No		Yes		No		Yes	
		N	%	N	%	N	%	N	%
Gender	Female	1242	52.4%	38	15.0%	887	42.9%	29	12.9%
	Male	1128	47.6%	216	85.0%	1181	57.1%	195	87.1%
Age group	<25	348	14.7%	91	35.8%	502	24.3%	111	49.6%
	25 ≤ age < 45	1246	52.6%	121	47.6%	1008	48.7%	98	43.8%
	45 ≤ age < 60	550	23.2%	34	13.4%	416	20.1%	13	5.8%
	60≤	226	9.5%	8	3.1%	142	6.9%	2	0.9%
Having driving license	No	569	24.0%	81	31.9%	1095	53.0%	131	58.4%
	Yes	1801	76.0%	173	68.1%	973	47.00%	93	41.6%
Having household car	Yes	2118	89.4%	209	82.3%	1457	70.5%	131	58.5%
	No	252	10.6%	45	17.7%	611	29.5%	93	41.5%
		Mean	Median	Mean	Median	Mean	Median	Mean	Median
Age		38.76	37.00	31.35	28.00	35.96	33.00	26.56	25.00
Household income (Euros) ^1^		1315.99	1169.00	1435.61	1169.00	7143,68	6000.00	6918.42	5000.00
Household income (country currency)		55,271,580 ^3^	49,098,000 ^3^	60,295,620 ^3^	49,098,000 ^3^	150,017.28 ^2^	126,000 ^2^	145,286.82 ^2^	105,000 ^2^
Monthly living cost (Euros) ^1^		1048.36	935.00	1302.18	935.00	6401.05	5500.00	6192.66	5000.00
Monthly living cost (country currency)		44,031,120^3^	39,270,000 ^3^	54,691,560 ^3^	39,270,000 ^3^	134,422.05 ^2^	1,155,000 ^2^	130,045.86 ^2^	105,000 ^2^

^1^ The amount is converted into EUR based on the central bank’s exchange rates in 2017 in Egypt and Iran, ^2^ Egyptian pound (Egyptian currency), ^3^ Iranian rial (Iranian Currency).

**Table 2:** The results of the Omnibus test and the Hosmer and Lemeshow test for biking.

Tests	Cairo	Tehran
Omnibus Tests of Model Coefficients		
Chi-square	233.955	274.906
*p*-value	<0.001	<0.001
−2 Log likelihood	1121.058	1281.290
Nagelkerke R Square	0.221	0.224
Hosmer and Lemeshow Test		
Chi-square	4.850	13.645
*p*-value	0.773	0.092

**Table 3:** Binary logit regression for cycling to near destination in Tehran.

Tehran		B	S.E.	Wald	AME	S.E.	Sig.	Exp(B)
Ridesourcing use	Regular users = 1 Non-regulars = 0	0.833	0.373	4.892	0.0605	0.0276	0.026	2.301
Gender	Female = 1. Male = 0	−2.057	0.196	110.183	−0.1494	0.0171	<0.001	0.128
Monthly household Income	Iranian rial	0.000	0.000	2.8614	0.000	0.000	0.091	1.000
Having a household car	No = 1. Yes = 0	0.324	0.217	2.227	0.0236	0.0155	0.136	1.383
Age	Year	−0.058	0.006	84.497	−0.0042	0.0005	<0.001	0.944
Link–node ratio	%	0.025	0.005	24.597	0.0018	0.0004	<0.001	1.025
Intersection density	Node/hectare	0.130	0.044	8.495	0.0094	0.0032	0.004	1.138
Constant		−4.123	0.927	19.801			<0.001	0.016

AME: Average Marginal Effects, S.E.: Standard Error.

**Table 4:** Binary logit regression for cycling to near destination in Cairo.

Cairo		B	S.E.	Wald	AME	S.E.	Sig.	Exp(B)
Ridesourcing use	Regular users = 1 Non-regulars = 0	0.663	0.302	4.815	0.0531	0.0234	0.028	1.940
Gender	Female = 1, Male = 0	−1.991	0.221	81.261	−0.1542	0.0172	<0.001	0.137
Monthly household Income	Egyptian pound	0.000	0.000	0.003	0.000	0.000	0.953	1.000
Having a household car	No = 1, Yes = 0	0.894	0.181	24.431	0.0692	0.0139	<0.001	2.445
Age	Year	−0.08	0.009	81.777	−0.0062	0.0007	<0.001	0.924
Link–node ratio	%	0.010	0.008	1.721	0.0008	0.0006	0.190	1.010
Intersection density	Node/hectare	−0.026	0.061	0.188	−0.0020	0.0047	0.665	0.974
Constant		−1.130	1.402	0.633			0.426	0.323

AME: Average Marginal Effects, S.E.: Standard Error.
